# Supplementary material for: Genome-wide specificity of dCpf1 cytidine base editors
Source: Nat Commun. 2020 Aug 13;11:4072. doi: 10.1038/s41467-020-17889-9 (PMC7426837; doi:10.1038/s41467-020-17889-9)
Supplement: Supplementary file 1 — Supplementary Information [file 41467_2020_17889_MOESM1_ESM.pdf]

Supplementary Information

Genome-wide specificity of dCpf1 cytidine base editors

Table of Contents

Supplemental Figures

Supplementary Figure 1. On-target activity of dLbCpf1-BE and LbCpf1.

Supplementary Figure 2. Mismatch tolerance of dLbCpf1-BE and LbCpf1 at the *DNMT1* and *EMX1* sites.

Supplementary Figure 3. Comparison of the results from two modified Digenome-seq experiments using dLbCpf1-BE with crRNA targeting *DYRK1A*.

Supplementary Figure 4. Comparison of in vitro cleavage sites induced by dLbCpf1-BE and LbCpf1.

Supplementary Figure 5. Base editing frequencies of dLbCpf1-BE and indel frequencies of LbCpf1 at 29 validated sites originally identified by dLbCpf1-BE-mediated Digenome-seq.

Supplementary Figure 6. Weak correlations between the ratio of the count of sequence reads with the same 5' end to the read depth (Count / Depth) in *in vitro* Digenome-seq results and dLbCpf1-mediated base editing frequencies in HEK293T cells at 29 validated sites.

Supplementary Figure 7. Base editing frequencies of dLbCpf1-BE with truncated or extended crRNAs.

Supplementary Figure 8. Base editing frequencies of dLbCpf1-BE containing mutations in the Cpf1 domain.

Supplementary Figure 9. Base editing frequencies of dLbCpf1-BE containing mutations in the APOBEC1 domain.

Supplemental Tables

Supplementary Table 1. The number of off-target candidates depending on the number of mismatches.

Supplementary Table 2. *In vitro* cleavage sites captured by dLbCpf1-BE-mediated Digenome-seq.

Supplementary Table 3. Number of *in vitro* cleavage sites identified by dLbCpf1-BE-mediated Digenome-seq.

Supplementary Table 4. Base editing frequencies of dLbCpf1-BE and Indel frequencies of LbCpf1 at off-target sites captured by dLbCpf1-BE-mediated Digenome-seq.

Supplementary Table 5. Base editing frequencies at off-target sites captured by LbCpf1 but not dLbCpf1-BE-mediated Digenome-seq.

Supplementary Table 6. OTI of dLbCpf1-BE.

Supplementary Table 7. Number of predicted off-target sites, defined as those sites with a PAM sequence (5'-TTTN-3') and up to eight mismatches with the target site, identified using Cas-OFFinder.

Supplementary Table 8. List of primers used for nested PCR.

Supplementary Figure 1. On-target activity of dLbCpf1-BE and LbCpf1.

(a,b) dLbCpf1-BE and LbCpf1 activities were tested at 9 human target sites in HEK293T cells. Base editing frequencies and indel frequencies were measured using targeted deep sequencing. Data are shown as mean ± s.e.m. (n = 9) (c) Scatterplots showing correlations between the dLbCpf1-mediated base editing frequency and the LbCpf1-mediated indel frequency at 9 human target sites.

a

					Base editing frequency (%)		Indel frequency (%)	
	PAM	target sequence	Chr	Location	Untreated	dLbCpf1-BE	Untreated	LbCpf1
CDKN2A	TTTA	GCCCCAATAATCCCCACATGTCA	chr9	21918616-21918638	0.14	26.85	0.06	50.11
RUNX	TTTC	TTCTCCCCTCTGCTGGATACCTC	chr21	36421323-36421345	0.14	30.81	0.00	68.87
FANCF	TTTA	TCCGTGTTCCTTGACTCTGGGCA	chr11	22647494-22647516	0.18	6.46	0.02	37.29
EMX1	TTTG	TCCTCCGGTTCTGGAACCACACC	chr2	73160921-73160943	0.06	2.35	0.03	30.57
DNMT1	TTTC	CTGATGGTCCATGTCTGTTACTC	chr19	10244443-10244465	0.76	14.71	0.02	51.47
LINC01551	TTTA	ATAATACACTCTTTACACTGAGG	chr14	29263957-29263979	0.26	13.62	0.00	44.42
DYRK1A	TTTA	GAAGCACATCAAGGACATTCTAA	chr21	38860230-38860252	0.08	39.49	0.01	62.18
BCL2L13	TTTA	ATTTCCAAGTCAACCTTATGAGG	chr22	18161369-18161391	0.13	3.09	0.00	24.08
CLIC4	TTTA	CCCTGGCTACCTCCCCTACCCGG	chr1	25167626-25167648	0.25	4.83	0.00	58.21

b

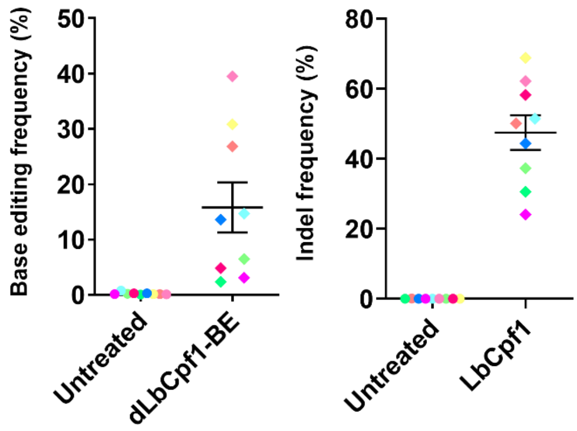

c

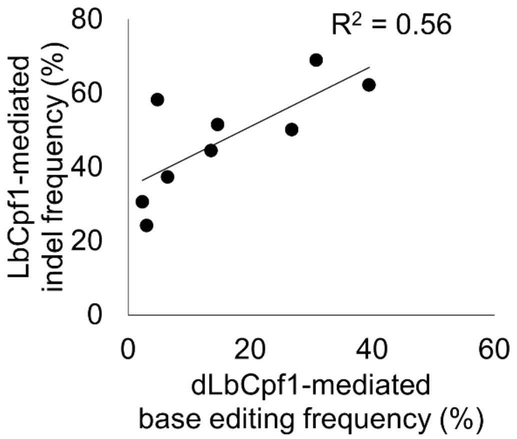

Supplementary Figure 2. Mismatch tolerance of dLbCpf1-BE and LbCpf1 at the *DNMT1* and *EMX1* sites.

(a,b) Plasmids encoding matched and mismatched crRNAs, containing 1- to 4-nt mismatches relative to the on-target site in *DNMT1* (a) and *EMX1* (b), were transfected into HEK293T cells with plasmids encoding dLbCpf1-BE or LbCpf1. Base editing frequencies and indel frequencies were measured using targeted deep sequencing. Mismatched bases and PAM sequences are shown in red and blue, respectively. The relative activity (the editing frequency with the mismatched crRNA divided by that with the matched crRNA) indicates the mismatch tolerance. Mismatched crRNAs for which the relative activity of dLbCpf1-BE is at least three times higher than that of LbCpf1 are indicated with blue asterisks; those for which the relative activity of LbCpf1 is at least three times higher are indicated with red asterisks. Data are shown as mean ± s.e.m. from three biologically independent samples. Source data are provided as a Source Data file.

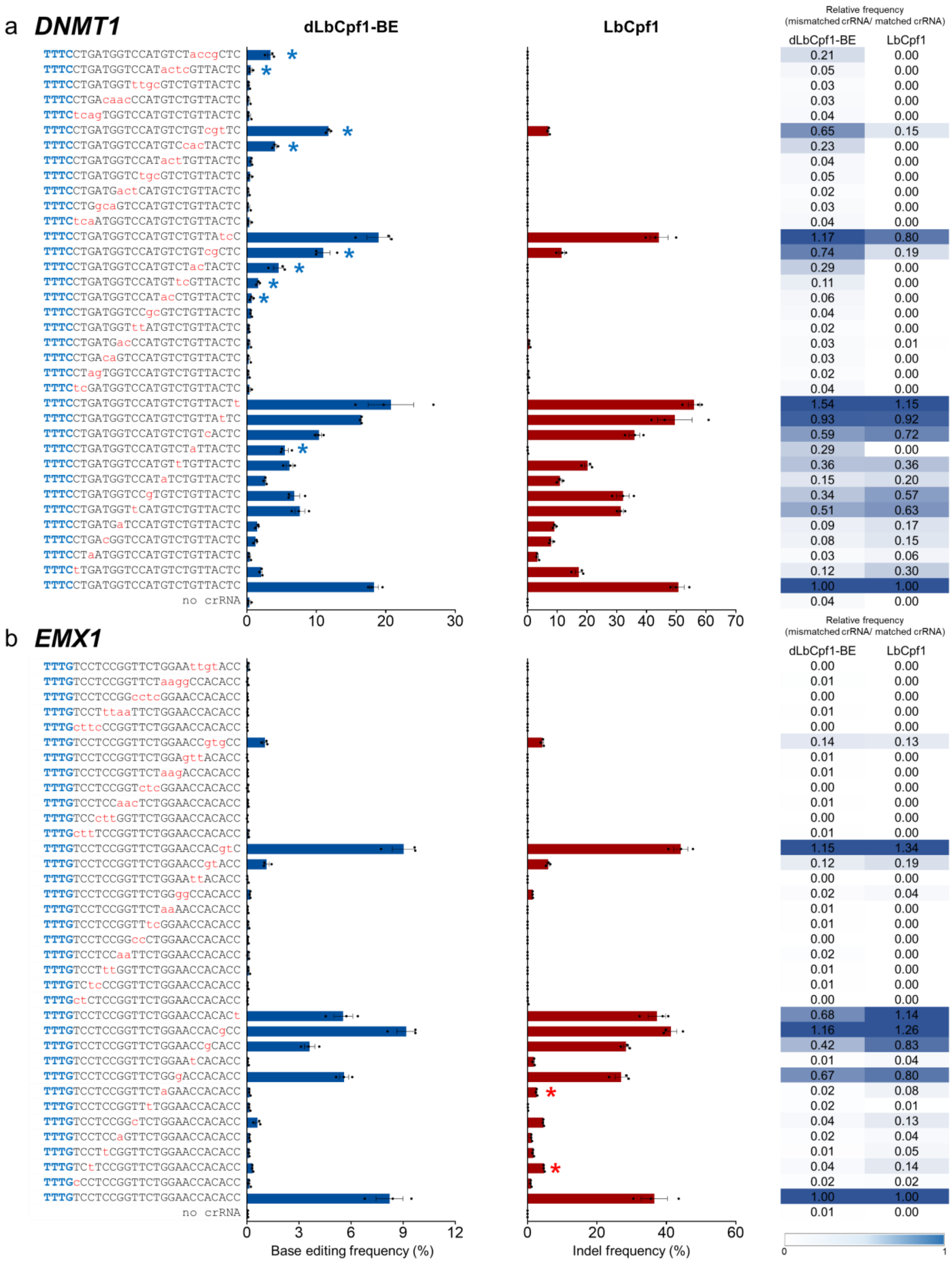

a
DNMT1
dLbCpf1-BE
LbCpf1
Relative frequency (mismatched crRNA/ matched crRNA)
dLbCpf1-BE
LbCpf1
TTTCCTGATGGTCCATGTCTaccgCTC
TTTCCTGATGGTCCATactcGTTACTC
TTTCCTGATGGTttgcGTCTGTTACTC
TTTCCTGAcaacCCATGTCTGTTACTC
TTTCtcagTGGTCCATGTCTGTTACTC
TTTCCTGATGGTCCATGTCTGTcgtTC
TTTCCTGATGGTCCATGTCcacTACTC
TTTCCTGATGGTCCATactTGTTACTC
TTTCCTGATGGTCtgcGTCTGTTACTC
TTTCCTGATGactCATGTCTGTTACTC
TTTCCTGgcaGTCCATGTCTGTTACTC
TTTCtcaATGGTCCATGTCTGTTACTC
TTTCCTGATGGTCCATGTCTGTTAtcC
TTTCCTGATGGTCCATGTCTGTcgCTC
TTTCCTGATGGTCCATGTCTacTACTC
TTTCCTGATGGTCCATGTtcGTTACTC
TTTCCTGATGGTCCATacCTGTTACTC
TTTCCTGATGGTCCgcGTCTGTTACTC
TTTCCTGATGGTttATGTCTGTTACTC
TTTCCTGATGacCCATGTCTGTTACTC
TTTCCTGAcaGTCCATGTCTGTTACTC
TTTCCTagTGGTCCATGTCTGTTACTC
TTTCtcGATGGTCCATGTCTGTTACTC
TTTCCTGATGGTCCATGTCTGTTACTt
TTTCCTGATGGTCCATGTCTGTTAtTC
TTTCCTGATGGTCCATGTCTGTcACTC
TTTCCTGATGGTCCATGTCTaTTACTC
TTTCCTGATGGTCCATGTtTGTTACTC
TTTCCTGATGGTCCATaTCTGTTACTC
TTTCCTGATGGTCCgTGTCTGTTACTC
TTTCCTGATGGTtCATGTCTGTTACTC
TTTCCTGATGaTCCATGTCTGTTACTC
TTTCCTGAcGGTCCATGTCTGTTACTC
TTTCCTaATGGTCCATGTCTGTTACTC
TTTCtTGATGGTCCATGTCTGTTACTC
TTTCCTGATGGTCCATGTCTGTTACTC
no crRNA
0
10
20
30
0
10
20
30
40
50
60
70
0.21 0.00
0.05 0.00
0.03 0.00
0.03 0.00
0.04 0.00
0.65 0.15
0.23 0.00
0.04 0.00
0.05 0.00
0.02 0.00
0.03 0.00
0.04 0.00
1.17 0.80
0.74 0.19
0.29 0.00
0.11 0.00
0.06 0.00
0.04 0.00
0.02 0.00
0.03 0.01
0.03 0.00
0.02 0.00
0.04 0.00
1.54 1.15
0.93 0.92
0.59 0.72
0.29 0.00
0.36 0.36
0.15 0.20
0.34 0.57
0.51 0.63
0.09 0.17
0.08 0.15
0.03 0.06
0.12 0.30
1.00 1.00
0.04 0.00
b
EMX1
Relative frequency (mismatched crRNA/ matched crRNA)
dLbCpf1-BE
LbCpf1
TTTGTCCTCCGGTTCTGGAAttgtACC
TTTGTCCTCCGGTTCTaaggCCACACC
TTTGTCCTCCGGcctcGGAACCACACC
TTTGTCCTttaaTTCTGGAACCACACC
TTTGcttcCCGGTTCTGGAACCACACC
TTTGTCCTCCGGTTCTGGAACCgtgCC
TTTGTCCTCCGGTTCTGGAgttACACC
TTTGTCCTCCGGTTCTaagACCACACC
TTTGTCCTCCGGTctcGGAACCACACC
TTTGTCCTCCaacTCTGGAACCACACC
TTTGTCCcttGGTTCTGGAACCACACC
TTTGcttTCCGGTTCTGGAACCACACC
TTTGTCCTCCGGTTCTGGAACCACgtC
TTTGTCCTCCGGTTCTGGAACCgtACC
TTTGTCCTCCGGTTCTGGAAttACACC
TTTGTCCTCCGGTTCTGGggCCACACC
TTTGTCCTCCGGTTCTaaAACCACACC
TTTGTCCTCCGGTTtcGGAACCACACC
TTTGTCCTCCGGccCTGGAACCACACC
TTTGTCCTCCaaTTCTGGAACCACACC
TTTGTCCTttGGTTCTGGAACCACACC
TTTGTCtcCCGGTTCTGGAACCACACC
TTTGctCTCCGGTTCTGGAACCACACC
TTTGTCCTCCGGTTCTGGAACCACACt
TTTGTCCTCCGGTTCTGGAACCACgCC
TTTGTCCTCCGGTTCTGGAACCgCACC
TTTGTCCTCCGGTTCTGGAAtCACACC
TTTGTCCTCCGGTTCTGGgACCACACC
TTTGTCCTCCGGTTCTaGAACCACACC
TTTGTCCTCCGGTTtTGGAACCACACC
TTTGTCCTCCGGcTCTGGAACCACACC
TTTGTCCTCCaGTTCTGGAACCACACC
TTTGTCCTtCGGTTCTGGAACCACACC
TTTGTCtTCCGGTTCTGGAACCACACC
TTTGcCCTCCGGTTCTGGAACCACACC
TTTGTCCTCCGGTTCTGGAACCACACC
no crRNA
0
3
6
9
12
Base editing frequency (%)
0
20
40
60
Indel frequency (%)
0.00 0.00
0.01 0.00
0.00 0.00
0.01 0.00
0.00 0.00
0.14 0.13
0.01 0.00
0.01 0.00
0.00 0.00
0.01 0.00
0.00 0.00
0.01 0.00
1.15 1.34
0.12 0.19
0.00 0.00
0.02 0.04
0.01 0.00
0.01 0.00
0.00 0.00
0.02 0.00
0.01 0.00
0.01 0.00
0.00 0.00
0.68 1.14
1.16 1.26
0.42 0.83
0.01 0.04
0.67 0.80
0.02 0.08
0.02 0.01
0.04 0.13
0.02 0.04
0.01 0.05
0.04 0.14
0.02 0.02
1.00 1.00
0.01 0.00
0
1

Supplementary Figure 3. Comparison of the results from two modified Digenome-seq experiments using dLbCpf1-BE with crRNA targeting *DYRK1A*.

Venn diagram showing the number of in vitro cleavage sites in the human genome identified by dLbCpf1-BE Digenome-seq at the *DYRK1A* target in two independent replicates.

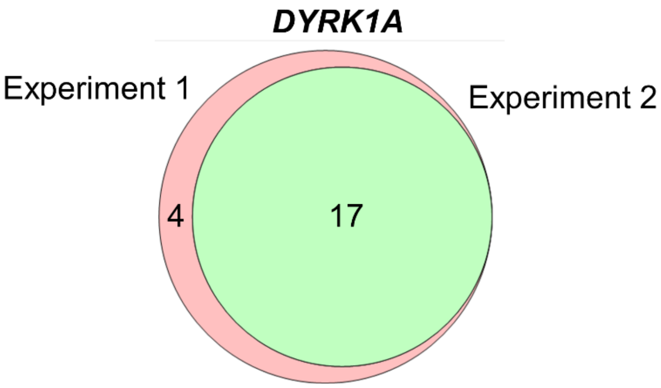

Supplementary Figure 4. Comparison of *in vitro* cleavage sites induced by dLbCpf1-BE and LbCpf1.

Representative Venn diagrams showing the *in vitro* cleavage sites identified by dLbCpf1-BE- and LbCpf1-mediated Digenome-seq at the *CDKN2A* (a) and *EMX1* (b) sites.

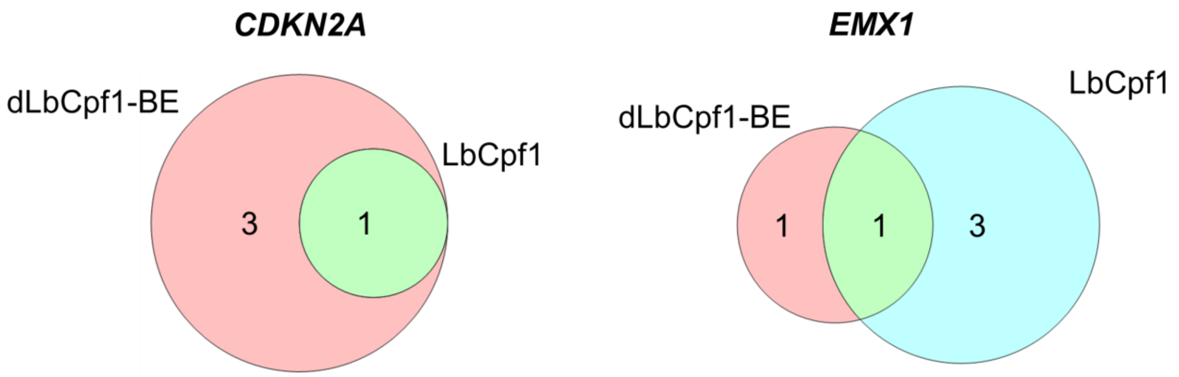

Supplementary Figure 5. Base editing frequencies of dLbCpf1-BE and indel frequencies of LbCpf1 at 29 validated sites originally identified by dLbCpf1-BE-mediated Digenome-seq.

Plasmids encoding crRNAs targeting nine endogenous genomic sites were individually transfected into HEK293T cells together with plasmids encoding dLBCpf1-BE or LbCpf1. Base editing frequencies of dLbCpf1-BE and indel frequencies of LbCpf1 were measured by targeted deep sequencing. This figure shows the 29 dLbCpf1-BE sites, a subset of the 106 candidate sites identified by dLbCpf1-BE-mediated Digenome-seq (listed in Supplementary Table 3), that were validated in HEK293T cells. Mismatched bases and PAM sequences are shown in red and blue, respectively. DNA and RNA bulge sequences are shown in green and with a dash, respectively. Data are shown as the mean of results from three biologically independent samples.

	Base editing frequency (%)		Indel frequency (%)	
	Untreated	(+)dLbCpf1-BE	Untreated	(+) LbCpf1
CDKN2A				
TTTAGCCCCAATcAaCtCtgCAct	0.03	0.09	0.02	0.01
TTTAGCCCCAATAATCCCCACATG	0.14	26.85	0.06	50.11
RUNX1				
TTTGTTCTCCCCTCTGCaGGATAa	0.46	9.03	0.19	1.66
TTTCTTCTCCCCTCTGCTatATAC	0.47	22.42	0.00	0.01
TTTCTTCTCCCCTCTGCaGGgcgg	0.37	12.60	0.00	0.00
TTTCTTCTCCCCTCTGCTGGATAC	0.14	30.81	0.00	68.87
FANCF				
TTTATCCGTGTTCCTTGACTCTGG	0.18	6.46	0.02	37.29
EMX1				
TTTGTCCTCCGGTTCTGGAACCAC	0.06	2.35	0.03	30.57
DNMT1				
TTTGCTGATGGTCtATagCTaTcA	0.04	0.07	0.00	0.01
TTTCCTGATGGTCCATGTCTGaat	0.13	3.43	0.01	1.43
TTTGCTGATGGTCtgTaTCTGTgA	0.08	0.13	0.02	0.01
TTTCCTGATGGTCCATGTCTGTTA	0.76	14.71	0.02	51.47
LINC01551				
TTTAATAATACA-TCTcTACAtac	0.12	0.31	0.01	0.00
TTTAATAATACA-TCTTatttaac	0.19	0.38	0.01	0.02
TTTAATAATACACTCTaTAatCac	0.34	1.53	0.00	0.00
TTTCATAATACA-TCTTTAtgCat	0.49	0.63	0.03	0.02
TTTAATAATACACTCTaacCtgat	0.75	1.76	0.01	0.00
TTTAATAATACACTCTTTACACTG	0.26	13.62	0.00	44.42
DYRK1A				
TTTGGAAGCACATCAtatgtAaTC	0.09	1.12	0.01	0.01
TTTAGAAGCACATCtcatACATTt	0.15	0.98	0.07	0.04
TTTAGAAGCACAcTCtAatAggTt	0.29	3.38	0.01	0.01
TTTGGAAGCACATCtAtGAgcTag	0.11	0.87	0.01	0.00
TTTAGAAGCACATCctactacTTa	0.19	2.13	0.20	0.21
TTTAGAAGCACATCA-GGACAaTC	0.07	1.59	0.01	0.00
TTTGGAAGCACATCtttGAaATgt	0.08	0.26	0.10	0.11
TTTAGAAGCACATCAAGGACATTC	0.08	39.49	0.01	62.18
BCL2L13				
TTTAATTTCCAAGTCAACCTTATG	0.13	3.09	0.00	24.08
CLIC4				
TTTCCCCTGaCTctCcCCtCTAtC	0.27	0.88	0.00	0.00
TTTACCCTGGCTACCTCCCCTACC	0.25	4.83	0.00	58.21

0 1 10

Supplementary Figure 6. Weak correlations between the ratio of the count of sequence reads with the same 5' end to the read depth (Count / Depth) in *in vitro* Digenome-seq results and dLbCpf1-mediated base editing frequencies in HEK293T cells at 29 validated sites.

Scatterplots showing correlations between the ratio of the count of sequence reads with the same 5' end to the read depth (Count / Depth) in *in vitro* Digenome-seq results and the mean of dLbCpf1-mediated base editing frequencies in HEK293T cells at 29 validated sites. The dLbCpf1-mediated base editing frequencies in HEK293T cells were measured by targeted deep sequencing of three biologically independent samples.

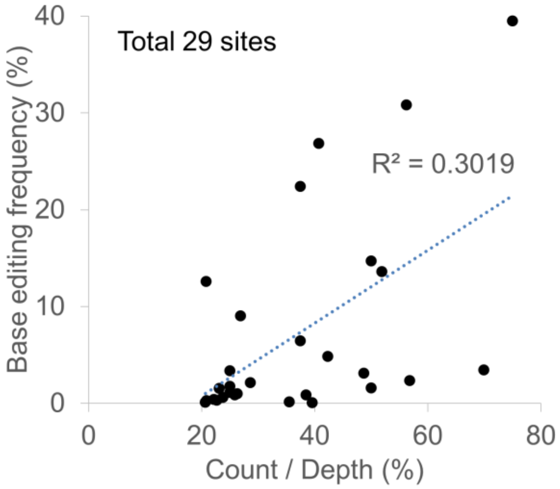

Supplementary Figure 7. Base editing frequencies of dLbCpf1-BE with truncated or extended crRNAs.

Plasmids encoding dLbCpf1-BE and truncated or extended crRNAs were transfected into HEK293T cells. Base editing frequencies at on- and -off target sites were measured via targeted deep sequencing. The specificity ratios were calculated by dividing (base editing frequency of dLbCpf1-BE with modified-length crRNA at on-target / that at off-target) by (base editing frequency of dLbCpf1-BE with a 23-nt crRNA at on-target / that at off-target). Mismatched bases and the PAM sequences are shown in red and blue, respectively. RNA bulges are represented as dashes and a DNA bulge is shown in green. Data are shown as mean ± s.e.m. from three biologically independent samples. Source data are provided as a Source Data file.

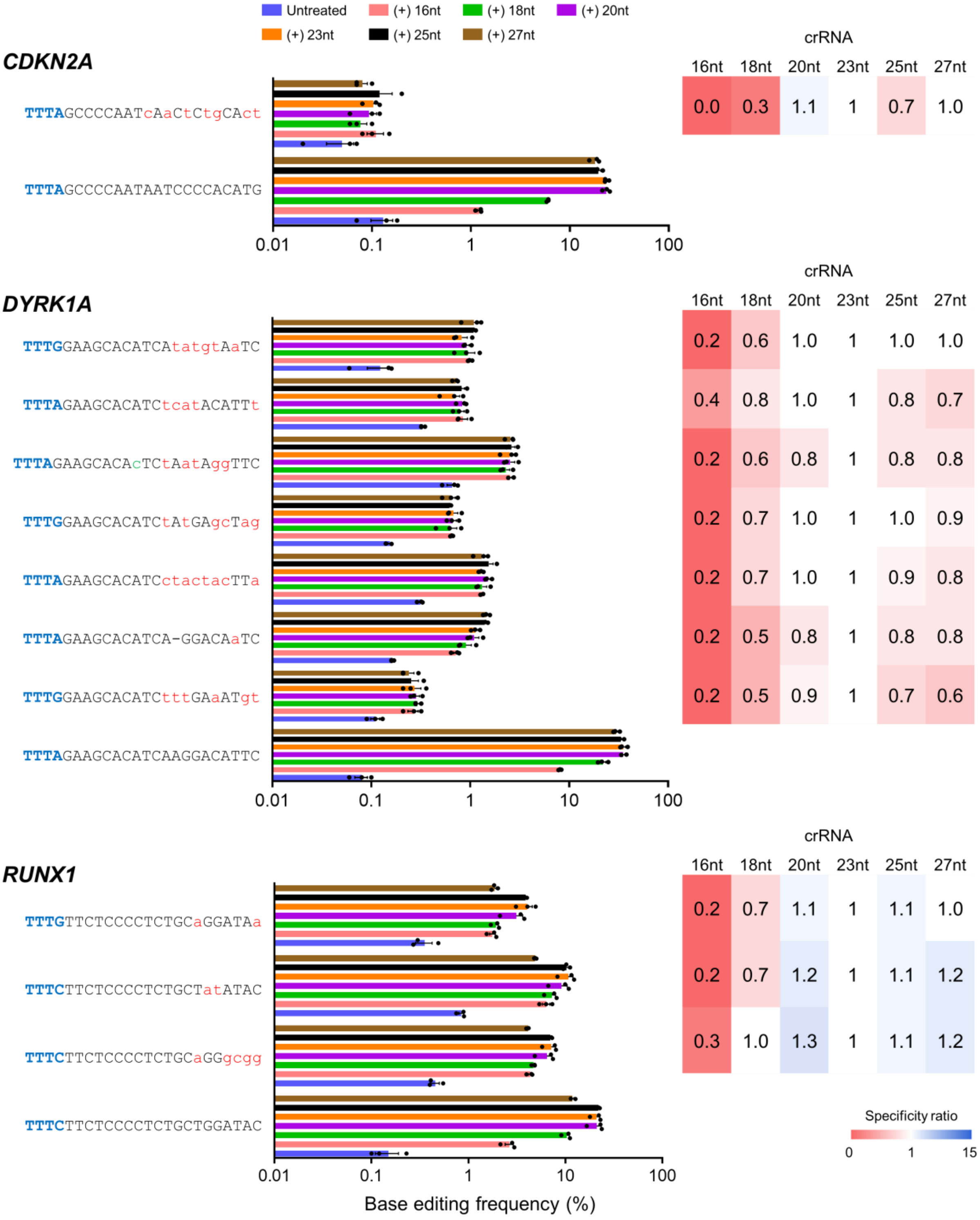
Untreated
(+) 16nt
(+) 18nt
(+) 20nt
(+) 23nt
(+) 25nt
(+) 27nt
CDKN2A
TTTAGCCCCAATcAaCtCtgCAct
TTTAGCCCCAATAATCCCCACATG
0.01
0.1
1
10
100
crRNA
16nt 18nt 20nt 23nt 25nt 27nt
0.0 0.3 1.1 1 0.7 1.0
DYRK1A
TTTGGAAGCACATCAtatgtAaTC
TTTAGAAGCACATCtcatACATTt
TTTAGAAGCACAcTCtAatAggTTC
TTTGGAAGCACATCtAtGAgcTag
TTTAGAAGCACATCctactacTTa
TTTAGAAGCACATCA-GGACAaTC
TTTGGAAGCACATCtttGAaATgt
TTTAGAAGCACATCAAGGACATTC
0.01
0.1
1
10
100
crRNA
16nt 18nt 20nt 23nt 25nt 27nt
0.2 0.6 1.0 1 1.0 1.0
0.4 0.8 1.0 1 0.8 0.7
0.2 0.6 0.8 1 0.8 0.8
0.2 0.7 1.0 1 1.0 0.9
0.2 0.7 1.0 1 0.9 0.8
0.2 0.5 0.8 1 0.8 0.8
0.2 0.5 0.9 1 0.7 0.6
RUNX1
TTTGTTCTCCCCTCTGCaGGATAa
TTTCTTCTCCCCTCTGCTatATAC
TTTCTTCTCCCCTCTGCaGGgcgg
TTTCTTCTCCCCTCTGCTGGATAC
0.01
0.1
1
10
100
Base editing frequency (%)
crRNA
16nt 18nt 20nt 23nt 25nt 27nt
0.2 0.7 1.1 1 1.1 1.0
0.2 0.7 1.2 1 1.1 1.2
0.3 1.0 1.3 1 1.1 1.2
Specificity ratio
0
1
15

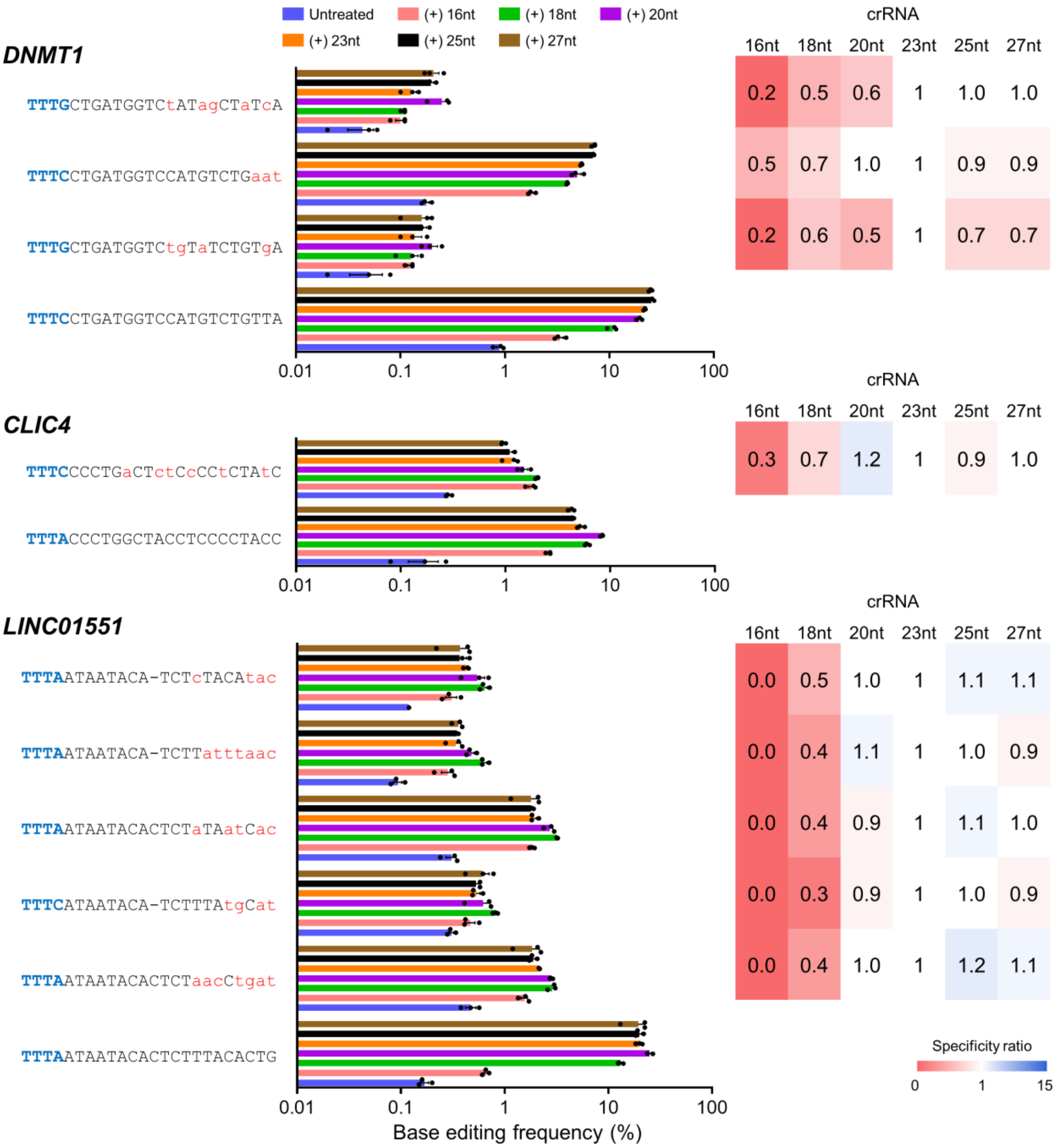

Untreated
(+) 16nt
(+) 18nt
(+) 20nt
(+) 23nt
(+) 25nt
(+) 27nt
DNMT1
TTTGCTGATGGTCtATagCTaTcA
TTTCCTGATGGTCCATGTCTGaat
TTTGCTGATGGTCtgTaTCTGTgA
TTTCCTGATGGTCCATGTCTGTTA
0.01
0.1
1
10
100
crRNA
16nt 18nt 20nt 23nt 25nt 27nt
0.2 0.5 0.6 1 1.0 1.0
0.5 0.7 1.0 1 0.9 0.9
0.2 0.6 0.5 1 0.7 0.7
CLIC4
TTTCCCCTGaCTctCcCCtCTAtC
TTTACCCTGGCTACCTCCCCTACC
0.01
0.1
1
10
100
crRNA
16nt 18nt 20nt 23nt 25nt 27nt
0.3 0.7 1.2 1 0.9 1.0
LINC01551
TTTAATAATACA-TCTcTACAtac
TTTAATAATACA-TCTTatttaac
TTTAATAATACACTCTaTAatCac
TTTCATAATACA-TCTTTAtgCat
TTTAATAATACACTCTaacCtgat
TTTAATAATACACTCTTTACACTG
0.01
0.1
1
10
100
Base editing frequency (%)
crRNA
16nt 18nt 20nt 23nt 25nt 27nt
0.0 0.5 1.0 1 1.1 1.1
0.0 0.4 1.1 1 1.0 0.9
0.0 0.4 0.9 1 1.1 1.0
0.0 0.3 0.9 1 1.0 0.9
0.0 0.4 1.0 1 1.2 1.1
Specificity ratio
0
1
15

Supplementary Figure 8. Base editing frequencies of dLbCpf1-BE containing mutations in the Cpf1 domain.

Plasmids encoding versions of dLbCpf1-BE containing mutations in the Cpf1 domain (N256A, N260A, S348A, K514A, K881A, or K897A) were transfected into HEK293T cells. Base editing frequencies at on- and -off target sites were measured using targeted deep sequencing. The specificity ratios were calculated by dividing (base editing frequency of dLbCpf1-BE variants at on-target / that at off-target) by (base editing frequency of dLbCpf1-BE-WT at on-target / that at off-target). Mismatched bases and PAM sequences are shown in red and blue, respectively. RNA bulges are represented as dashes and a DNA bulge is shown in green. Data are shown as mean ± s.e.m. from three biologically independent samples. Source data are provided as a Source Data file.

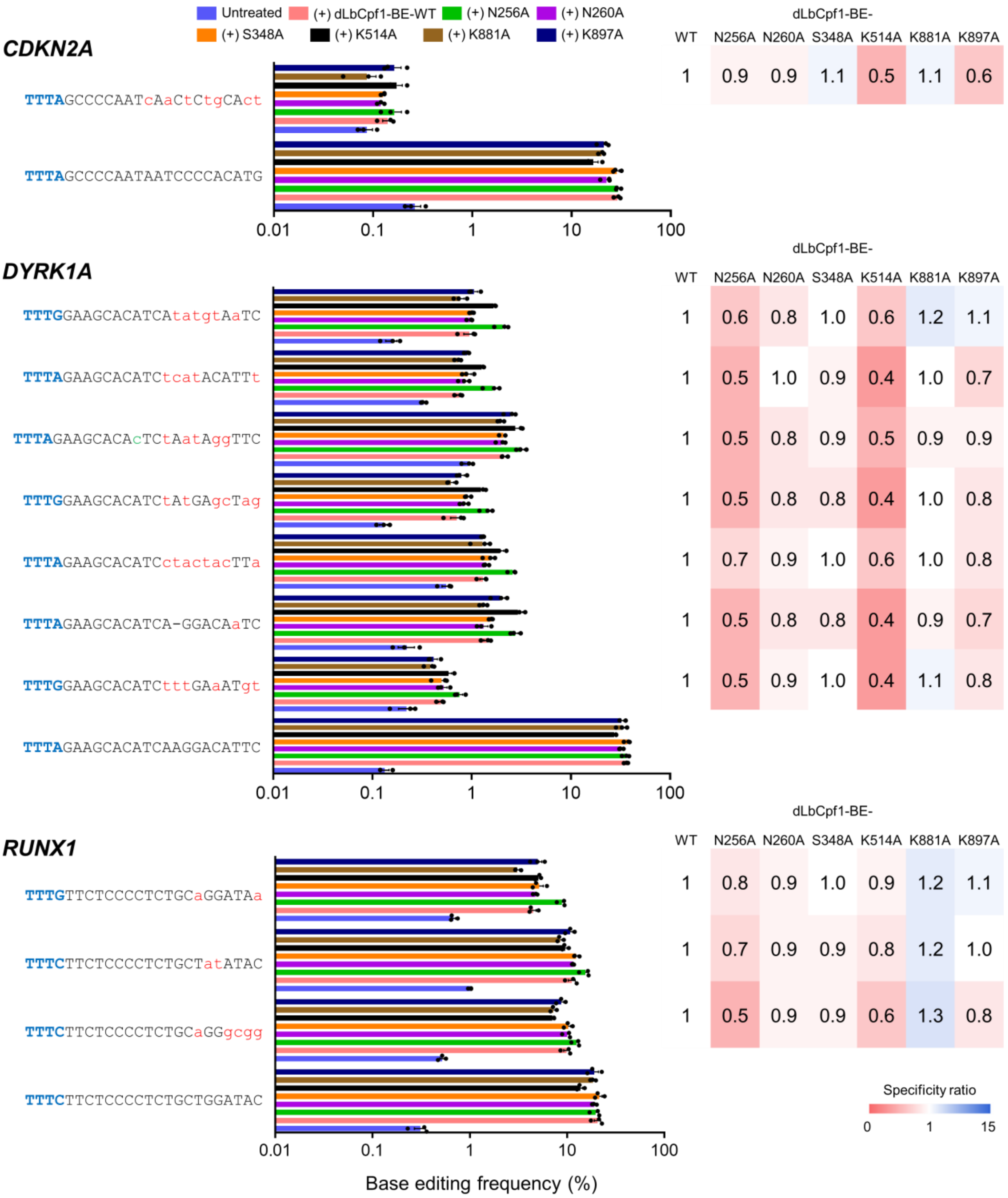

Untreated
(+) dLbCpf1-BE-WT
(+) N256A
(+) N260A
(+) S348A
(+) K514A
(+) K881A
(+) K897A
CDKN2A
TTTAGCCCCAATcAaCtCtgCAct
TTTAGCCCCAATAATCCCCACATG
0.01 0.1 1 10 100
dLbCpf1-BE-
WT N256A N260A S348A K514A K881A K897A
1 0.9 0.9 1.1 0.5 1.1 0.6
DYRK1A
TTTGGAAGCACATCAtatgtAaTC
TTTAGAAGCACATCtcatACATTt
TTTAGAAGCACAcTCtAatAggTTC
TTTGGAAGCACATCtAtGAgcTag
TTTAGAAGCACATCctactacTTa
TTTAGAAGCACATCA-GGACAaTC
TTTGGAAGCACATCtttGAaATgt
TTTAGAAGCACATCAAGGACATTC
0.01 0.1 1 10 100
dLbCpf1-BE-
WT N256A N260A S348A K514A K881A K897A
1 0.6 0.8 1.0 0.6 1.2 1.1
1 0.5 1.0 0.9 0.4 1.0 0.7
1 0.5 0.8 0.9 0.5 0.9 0.9
1 0.5 0.8 0.8 0.4 1.0 0.8
1 0.7 0.9 1.0 0.6 1.0 0.8
1 0.5 0.8 0.8 0.4 0.9 0.7
1 0.5 0.9 1.0 0.4 1.1 0.8
RUNX1
TTTGTTCTCCCCTCTGCaGGATAa
TTTCTTCTCCCCTCTGCTatATAC
TTTCTTCTCCCCTCTGCaGGgcgg
TTTCTTCTCCCCTCTGCTGGATAC
0.01 0.1 1 10 100
Base editing frequency (%)
dLbCpf1-BE-
WT N256A N260A S348A K514A K881A K897A
1 0.8 0.9 1.0 0.9 1.2 1.1
1 0.7 0.9 0.9 0.8 1.2 1.0
1 0.5 0.9 0.9 0.6 1.3 0.8
Specificity ratio
0 1 15

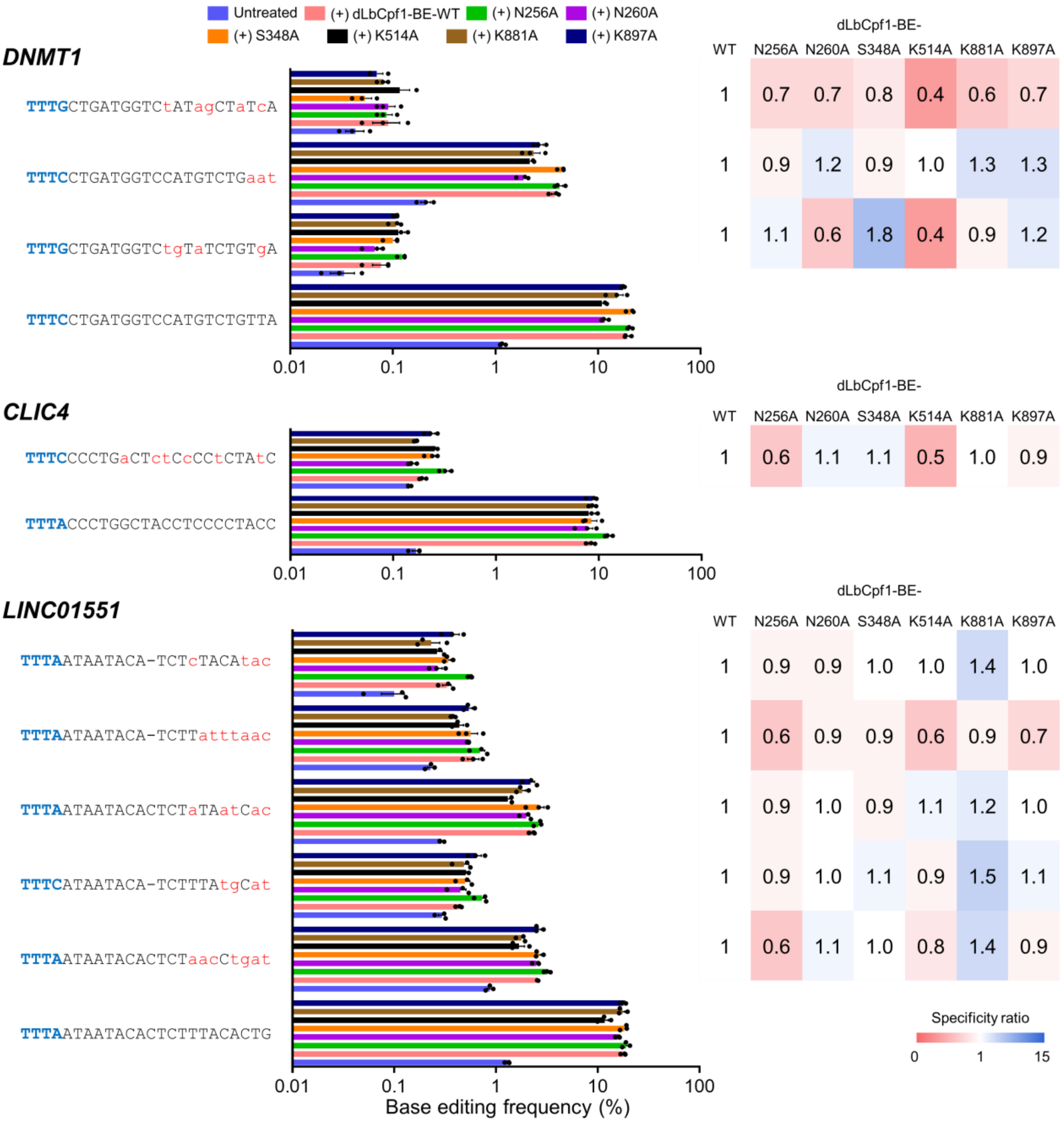
Untreated
(+) dLbCpf1-BE-WT
(+) N256A
(+) N260A
(+) S348A
(+) K514A
(+) K881A
(+) K897A
DNMT1
TTTGCTGATGGTCtATagCTaTcA
TTTCCTGATGGTCCATGTCTGaat
TTTGCTGATGGTCtgTaTCTGTgA
TTTCCTGATGGTCCATGTCTGTTA
0.01 0.1 1 10 100
dLbCpf1-BE-
WT N256A N260A S348A K514A K881A K897A
1 0.7 0.7 0.8 0.4 0.6 0.7
1 0.9 1.2 0.9 1.0 1.3 1.3
1 1.1 0.6 1.8 0.4 0.9 1.2
CLIC4
TTTCCCCTGaCTctCcCCtCTAtC
TTTACCCTGGCTACCTCCCCTACC
0.01 0.1 1 10 100
dLbCpf1-BE-
WT N256A N260A S348A K514A K881A K897A
1 0.6 1.1 1.1 0.5 1.0 0.9
LINC01551
TTTAATAATACA-TCTcTACAtac
TTTAATAATACA-TCTTatttaac
TTTAATAATACACTCTaTAatCac
TTTCATAATACA-TCTTTAtgCat
TTTAATAATACACTCTaacCtgat
TTTAATAATACACTCTTTACACTG
0.01 0.1 1 10 100
Base editing frequency (%)
dLbCpf1-BE-
WT N256A N260A S348A K514A K881A K897A
1 0.9 0.9 1.0 1.0 1.4 1.0
1 0.6 0.9 0.9 0.6 0.9 0.7
1 0.9 1.0 0.9 1.1 1.2 1.0
1 0.9 1.0 1.1 0.9 1.5 1.1
1 0.6 1.1 1.0 0.8 1.4 0.9
Specificity ratio
0 1 15

Supplementary Figure 9. Base editing frequencies of dLbCpf1-BE containing mutations in the APOBEC1 domain.

Plasmids encoding versions of dLbCpf1-BE containing mutations in the APOBEC1 domain (YE1: W90Y+R126E; YE2: W90Y+R132E; EE: R126E+R132E; YEE: W90Y+R126E+R132E) were transfected into HEK293T cells. Base editing frequencies at on- and -off target sites were measured using targeted deep sequencing. The specificity ratios were calculated by dividing (base editing frequency of dLbCpf1-BE variants at on-target / that at off-target) by (base editing frequency of dLbCpf1-BE-WT at on-target / that at off-target). Mismatched bases and PAM sequences are shown in red and blue, respectively. RNA bulges are represented as dashes and a DNA bulge is shown in green. Data are shown as mean ± s.e.m. from three biologically independent samples. Source data are provided as a Source Data file.

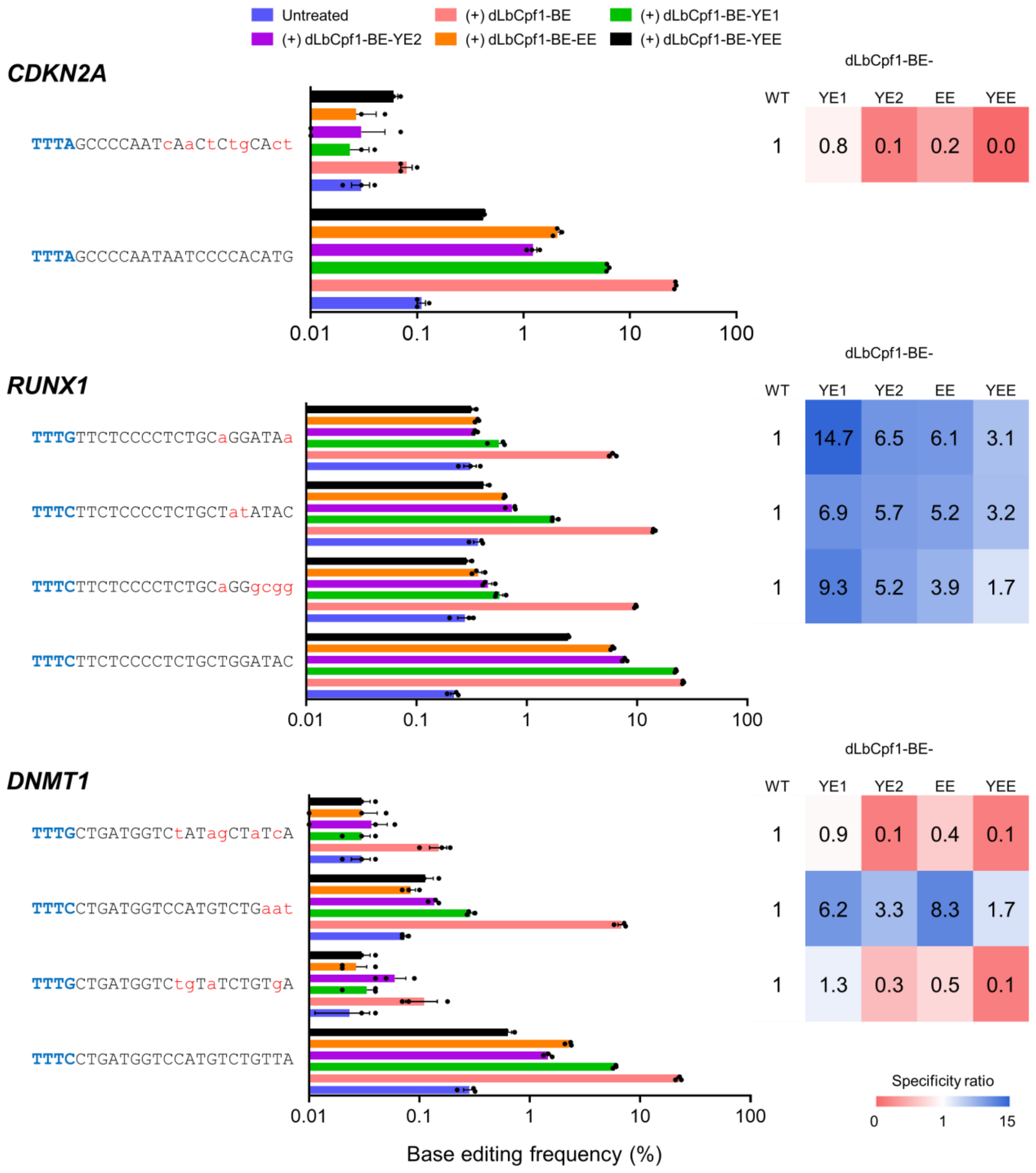
Untreated
(+) dLbCpf1-BE
(+) dLbCpf1-BE-YE1
(+) dLbCpf1-BE-YE2
(+) dLbCpf1-BE-EE
(+) dLbCpf1-BE-YEE
CDKN2A
TTTAGCCCCAATcAaCtCtgCAct
TTTAGCCCCAATAATCCCCACATG
0.01
0.1
1
10
100
dLbCpf1-BE-
WT YE1 YE2 EE YEE
1 0.8 0.1 0.2 0.0
RUNX1
TTTGTTCTCCCCTCTGCaGGATAa
TTTCTTCTCCCCTCTGCTatATAC
TTTCTTCTCCCCTCTGCaGGgcgg
TTTCTTCTCCCCTCTGCTGGATAC
0.01
0.1
1
10
100
dLbCpf1-BE-
WT YE1 YE2 EE YEE
1 14.7 6.5 6.1 3.1
1 6.9 5.7 5.2 3.2
1 9.3 5.2 3.9 1.7
DNMT1
TTTGCTGATGGTCtATagCTaTcA
TTTCCTGATGGTCCATGTCTGaat
TTTGCTGATGGTCtgTaTCTGTgA
TTTCCTGATGGTCCATGTCTGTTA
0.01
0.1
1
10
100
Base editing frequency (%)
dLbCpf1-BE-
WT YE1 YE2 EE YEE
1 0.9 0.1 0.4 0.1
1 6.2 3.3 8.3 1.7
1 1.3 0.3 0.5 0.1
Specificity ratio
0
1
15

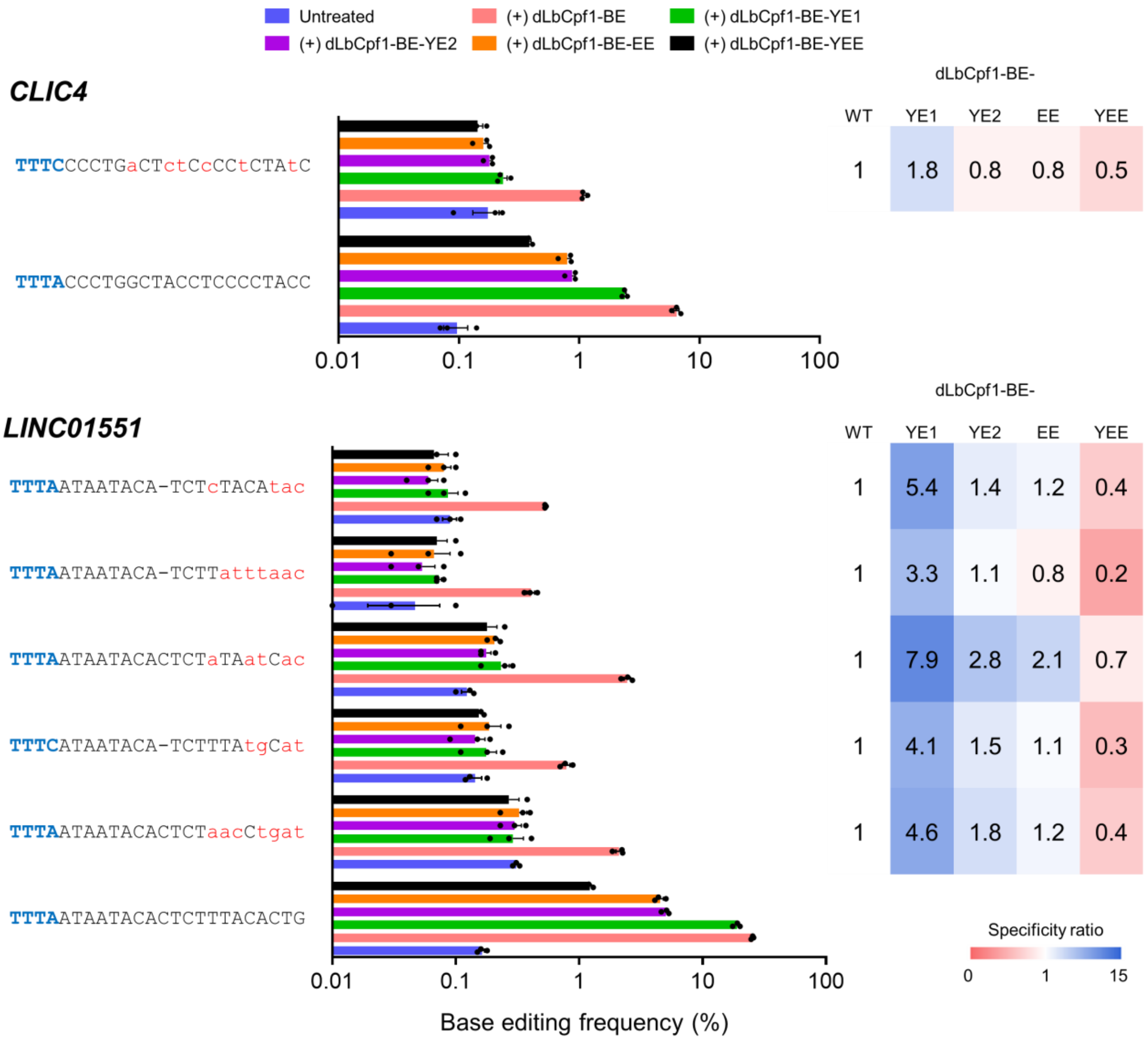

Untreated
(+) dLbCpf1-BE
(+) dLbCpf1-BE-YE1
(+) dLbCpf1-BE-YE2
(+) dLbCpf1-BE-EE
(+) dLbCpf1-BE-YEE
CLIC4
TTTCCCCTGaCTctCcCCtCTAtC
TTTACCCTGGCTACCTCCCCTACC
0.01
0.1
1
10
100
dLbCpf1-BE-
WT
YE1
YE2
EE
YEE
1
1.8
0.8
0.8
0.5
LINC01551
TTTAATAATACA-TCTcTACAtac
TTTAATAATACA-TCTTatttaac
TTTAATAATACACTCTaTAatCac
TTTCATAATACA-TCTTTAtgCat
TTTAATAATACACTCTaacCtgat
TTTAATAATACACTCTTTACACTG
Base editing frequency (%)
dLbCpf1-BE-
WT
YE1
YE2
EE
YEE
1
5.4
1.4
1.2
0.4
1
3.3
1.1
0.8
0.2
1
7.9
2.8
2.1
0.7
1
4.1
1.5
1.1
0.3
1
4.6
1.8
1.2
0.4
Specificity ratio
0
1
15
