# Supplementary material for: Genome-wide specificity of dCpf1 cytidine base editors
Source: Nat Commun. 2020 Aug 13;11:4072. doi: 10.1038/s41467-020-17889-9 (PMC7426837; doi:10.1038/s41467-020-17889-9)
Supplement: Supplementary file 3 — Reporting Summary [file 41467_2020_17889_MOESM3_ESM.pdf]

nature research

Corresponding author(s): Daesik Kim, Jin-Soo Kim

Last updated by author(s): Jul 2, 2020

Reporting Summary

Nature Research wishes to improve the reproducibility of the work that we publish. This form provides structure for consistency and transparency in reporting. For further information on Nature Research policies, see Authors & Referees and the Editorial Policy Checklist .

Statistics

For all statistical analyses, confirm that the following items are present in the figure legend, table legend, main text, or Methods section.

n/a	Confirmed	
☐	☒	The exact sample size (*n*) for each experimental group/condition, given as a discrete number and unit of measurement
☒	☐	A statement on whether measurements were taken from distinct samples or whether the same sample was measured repeatedly
☒	☐	The statistical test(s) used AND whether they are one- or two-sided<br>*Only common tests should be described solely by name; describe more complex techniques in the Methods section.*
☒	☐	A description of all covariates tested
☒	☐	A description of any assumptions or corrections, such as tests of normality and adjustment for multiple comparisons
☐	☒	A full description of the statistical parameters including central tendency (e.g. means) or other basic estimates (e.g. regression coefficient) AND variation (e.g. standard deviation) or associated estimates of uncertainty (e.g. confidence intervals)
☒	☐	For null hypothesis testing, the test statistic (e.g. *F*, *t*, *r*) with confidence intervals, effect sizes, degrees of freedom and *P* value noted<br>*Give P values as exact values whenever suitable.*
☒	☐	For Bayesian analysis, information on the choice of priors and Markov chain Monte Carlo settings
☒	☐	For hierarchical and complex designs, identification of the appropriate level for tests and full reporting of outcomes
☒	☐	Estimates of effect sizes (e.g. Cohen's *d*, Pearson's *r*), indicating how they were calculated

Our web collection on statistics for biologists contains articles on many of the points above.

Software and code

Policy information about availability of computer code

Data collection	Whole-genome sequencing was performed using the HiseqX sequencing system(Illumina) at Macrogen. PCR amplicons were deep sequenced using the mini-seq (Illumina) as paired end 150 reads
Data analysis	Isaac aligner(01.15.02.08), SAMtools (1.3.1), Prism 8, Digenome-toolkit (available at https://github.com/snugel/digenome-toolkit), and MAUND (available at https://github.com/ibs-cge/maund) was used in the data analysis.

For manuscripts utilizing custom algorithms or software that are central to the research but not yet described in published literature, software must be made available to editors/reviewers. We strongly encourage code deposition in a community repository (e.g. GitHub). See the Nature Research guidelines for submitting code & software for further information.

Data

Policy information about availability of data

All manuscripts must include a data availability statement. This statement should provide the following information, where applicable:

- Accession codes, unique identifiers, or web links for publicly available datasets
- A list of figures that have associated raw data
- A description of any restrictions on data availability

Sequencing data have been deposited in the NCBI Sequence Read Archive (SRA) database with BioProject accession code PRJNA630828 [https://www.ncbi.nlm.nih.gov/sra/?term=PRJNA630828] and PRJNA634784 [https://www.ncbi.nlm.nih.gov/sra/?term=PRJNA634784]

Field-specific reporting

Please select the one below that is the best fit for your research. If you are not sure, read the appropriate sections before making your selection.

Life sciences study design

All studies must disclose on these points even when the disclosure is negative.

Sample size	A total of 9 sites were identified by nDigenome-seq. Other experiments were performed in biological triplicate unless otherwise noted. In previous studies we determined this sample size to be sufficient to ensure reproducibility. Statistical methods were not used to decide sample size.
Data exclusions	No data were excluded from the analysis.
Replication	For each experiment, at least three independent repeats were performed. Results were comparable across these conditions.
Randomization	No randomization was necessary for cell based experiments because investigators were performed experiments under well controlled conditions.
Blinding	Investigators were not blinded during experiments and data analysis.

Reporting for specific materials, systems and methods

We require information from authors about some types of materials, experimental systems and methods used in many studies. Here, indicate whether each material, system or method listed is relevant to your study. If you are not sure if a list item applies to your research, read the appropriate section before selecting a response.

Materials & experimental systems

n/a	Involved in the study	
☒	☐	Antibodies
☐	☒	Eukaryotic cell lines
☒	☐	Palaeontology
☒	☐	Animals and other organisms
☒	☐	Human research participants
☒	☐	Clinical data

Methods

n/a	Involved in the study	
☒	☐	ChIP-seq
☒	☐	Flow cytometry
☒	☐	MRI-based neuroimaging

Eukaryotic cell lines

Policy information about cell lines

Cell line source(s)	HEK293T/17 cells obtained from ATCC
Authentication	No eukarytoic cell lined were authenticated.
Mycoplasma contamination	Cell lines were not tested mycoplasma contamination
Commonly misidentified lines (See ICLAC register)	No commonly misidentified cell lines were used.
